# Complex presentation of polyarteritis nodosa: Renal pseudoaneurysm rupture and bowel ischemia: A case report

**DOI:** 10.1016/j.ijscr.2025.111172

**Published:** 2025-03-18

**Authors:** Ali F. Rabaya, Kareem Ibraheem, Omar Shawer, Mutasem N. Nairat, Sayf Sayes, Rafiq Salhab

**Affiliations:** a Faculty of Medicine, Hebron University, Hebron, Palestine; b Faculty of Medicine, Palestine Polytechnic University, Hebron 90200, Palestine; c Palestinian Clinical Research Center, Bethlehem, Palestine; d Faculty of Medicine, Ain Shams University, Cairo, Egypt; e General Surgery Department, Ahli Hospital, Hebron, Palestine; Faculty of Medicine, Palestine Polytechnic University, Palestine; f General Surgery Department, Ahli Hospital, Hebron, Palestine

**Keywords:** Polyarteritis nodosa, Renal artery aneurysm, Gastrointestinal ischemia, Vasculitis, Superior mesenteric artery

## Abstract

**Introduction:**

Polyarteritis Nodosa (PAN) is a rare vasculitis of medium-sized arteries with severe complications, including Renal artery aneurysm (RAA)rupture and gastrointestinal ischemia. Early recognition and intervention are crucial for improving outcomes.

**Presentation of case:**

A 21-year-old male with a history of PAN presented with acute flank pain, rash, confusion, and anemia. Imaging revealed a ruptured RAA with retroperitoneal hemorrhage. He developed bowel ischemia confirmed by colonoscopy and computed tomography scan. The patient underwent renal artery embolization and exploratory laparotomy, revealing gangrenous cecum and ileal perforation, requiring hemicolectomy and ileal resection. Postoperatively, he was treated with immunoglobulin, cyclophosphamide, and prednisone, resulting in significant improvement.

**Discussion:**

PAN can cause life-threatening complications, including vascular rupture and ischemia. Embolization effectively controlled bleeding, and surgery addressed ischemic damage. Early intervention with immunosuppressive therapy played a key role in recovery.

**Conclusion:**

This case emphasizes the need for prompt recognition and treatment of severe PAN complications. Early surgical intervention and appropriate immunosuppressive therapy are essential for preventing fatal outcomes.

## Highlights

• A 21-year-old male with Polyarteritis Nodosa presented with renal and mesenteric aneurysms.
• Polyarteritis nodosa is a necrotizing vasculitis of medium-sized arteries, characterized by transmural inflammation, fibrinoid necrosis, and vascular weakening, predisposing to aneurysm formation.
• Renal and mesenteric aneurysms arise from arterial wall damage, increasing the risk of ischemia, rupture, and infarction.
• Histopathology reveals segmental arterial inflammation with neutrophilic infiltration, fibrinoid necrosis, and vascular congestion, confirming PAN diagnosis.
• Early immunosuppressive therapy with glucocorticoids and cyclophosphamide can prevent disease progression and improve outcomes.

## 1 Introduction

Classic polyarteritis nodosa (PAN) is a systemic necrotizing vasculitis that primarily affects medium and small muscular arteries, especially at branch points [1]. The estimated incidence of PAN ranges from 0 to 9 cases per million per year [2], with a male predominance and peak onset in the fourth to fifth decade of life. However, severe presentations in young adults are rare. PAN commonly involves the skin, joints, peripheral nerves, gastrointestinal tract, and kidneys [1]. Mesenteric ischemia occurs in up to 50 % of PAN cases, resulting from arterial inflammation, thrombosis, or aneurysmal rupture, leading to bowel infarction and perforation [3].

This study presents a severe case of PAN complicated by Renal artery aneurysm (RAA) rupture and bowel ischemia, emphasizing the importance of early recognition, clinical diagnosis, and aggressive treatment to improve patient outcomes.

## 2 Case presentation

This case report adheres to the SCARE 2023 guidelines for surgical case reporting [12].

**Fig. 1:**
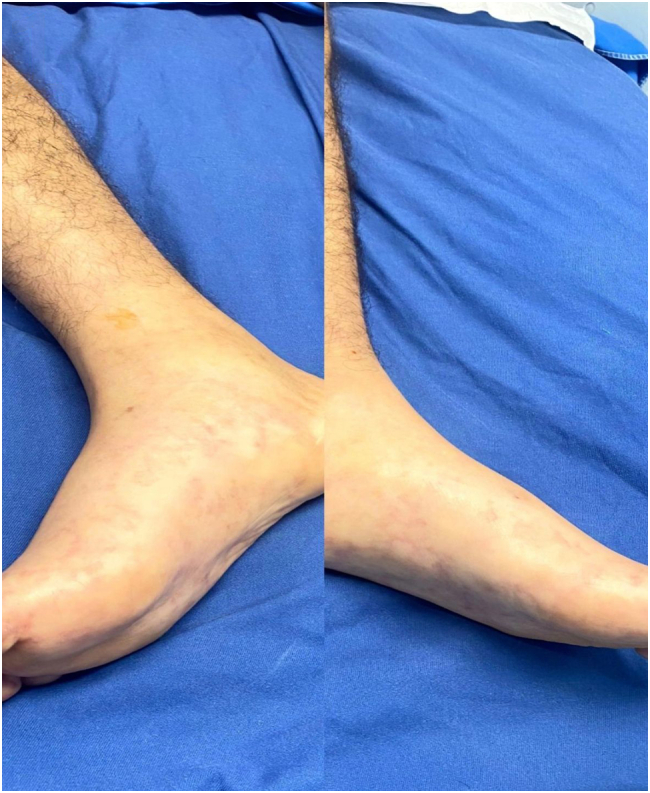
Livedo Reticularis. This is a mottled or lace-like rash that appears as a reddish or purplish on the lower legs.

A 21-year-old male patient with a known history of Polyarteritis Nodosa (PAN) for three years, previously managed with intermittent corticosteroid therapy and immunosuppressive agents, including methotrexate and azathioprine, with variable disease control, was admitted to our hospital on September 27, 2024, with complaints of right flank pain for one day, accompanied by a skin rash on the lower limbs (Fig. 1), chills, an acute confusional state, and a drop in hemoglobin to 7.6 g/dL. The patient had previously been in good health, with no significant past surgical history except for a varicocelectomy one year prior. His past medical management for PAN had not been well documented. On physical examination, the patient's temperature was 36.6 °C, blood pressure was 136/93 mmHg, and pain was rated at 5 out of 10. His pulse rate at presentation was 110 b/m and respiratory rate was 18/min, oxygen saturation 94 % on room air, BMI was 20 kg/m 2. An acute confusional state was noted. Cardiopulmonary auscultation did not reveal any abnormalities. The abdomen was flat and soft, with tenderness in the right lower quadrant and flank, but without rebound pain. The liver and spleen were not palpable below the costal margin, and bowel sounds were normal. The patient exhibited muscle tenderness and superficial hypoesthesia in both lower limbs, with muscle strength graded 5/5 in all four limbs.

A nasogastric (NG) probe was inserted upon admission to decompress the stomach, manage potential gastric distension, and assist in reducing the risk of aspiration, especially since the patient had an acute confusional state and was at risk for gastrointestinal motility issues. The NG probe was kept in place to monitor and manage any further gastrointestinal complications. An abdominal and pelvic CT scan with intravenous contrast was ordered, which revealed a rupture of a partially thrombosed RAA in the right renal mid-pole, possibly arising from an accessory artery to the right lower pole, along with acute retroperitoneal hemorrhage and active bleeding. Two aneurysmal dilatations, each measuring approximately 10 mm, were identified in the branches of the superior mesenteric artery (SMA) at the mid-anterior abdominal cavity, with one of them partially thrombosed. The patient subsequently underwent abdominal angiography and embolization of the accessory renal artery. In addition to embolization, the patient received packed red blood cell transfusions to address anemia resulting from the retroperitoneal hemorrhage, alongside close hemodynamic monitoring and supportive care. Fig. 2A shows the angiographic findings before coiling, and Fig. 2B shows the post-coiling result. Treatment for the retroperitoneal hemorrhage beyond embolization should be detailed, considering its contribution to anemia.

**Fig. 2A:**
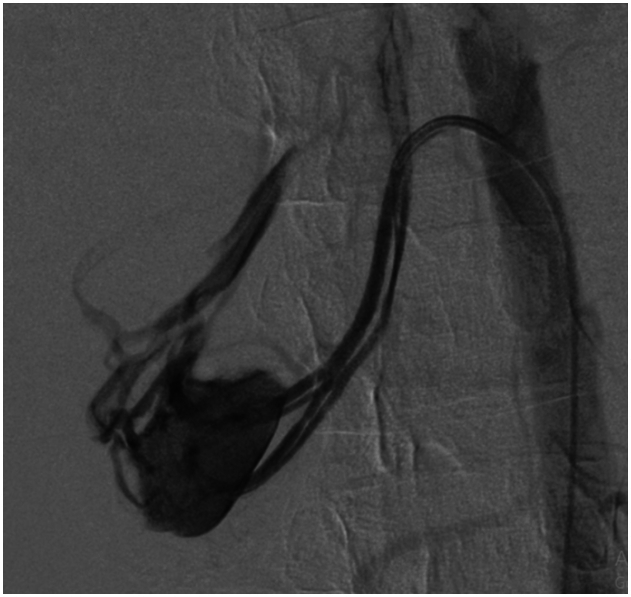
Angiographic findings show rupture of a partially thrombosed pseudoaneurysm in the right renal mid-pole arising from an accessory artery to the right lower pole.

**Fig. 2B:**
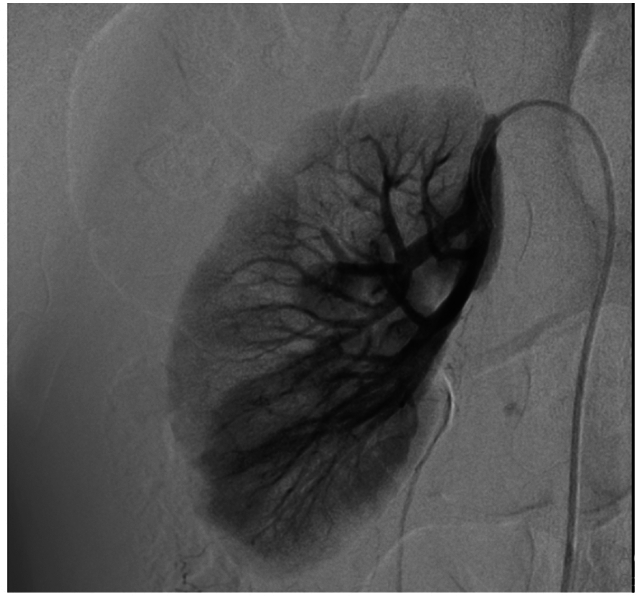
Angiography of the accessory renal artery after embolization.

On October 6, 2024, the patient began experiencing abdominal pain with bloody diarrhea. Blood tests indicated a leukocyte count of 11.80 × 10^9/L, hemoglobin of 10 g/dL, platelet count of 514 × 10^9/L, and an erythrocyte sedimentation rate (ESR) of 53 mm/h. Routine urine, biochemical, and coagulation tests were within normal limits. Hepatitis B and C antibodies were negative. C-reactive protein (CRP) was elevated at 111 mg/L, while tumor markers were negative. Stool examination and an occult blood test were positive for occult blood. Quantitative ANA, ANA profile, and ANCA tests were within normal ranges. An electrocardiogram revealed an incomplete right bundle branch block. Electromyography showed F-wave abnormalities in both lower limbs, suggesting potential proximal nerve or root damage, with suspected L5/S1 segmental involvement on the left side. A chest CT scan revealed no abnormalities. A colonoscopy was performed, revealing multiple anorectal ulcers with oozing blood and suspected gangrenous rectal mucosa; the procedure was terminated due to a high suspicion of bowel ischemia. Urgent surgical consultation was obtained.

**Fig. 3:**
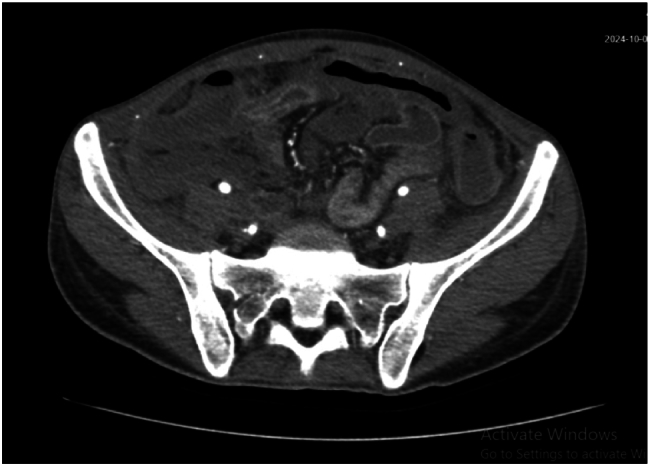
Abdominal CT revealed thickening of the colonic wall, increased density of surrounding mesenteric fat due to inflammation or edema, and mucosal layer enhancement.

**Fig. 4:**
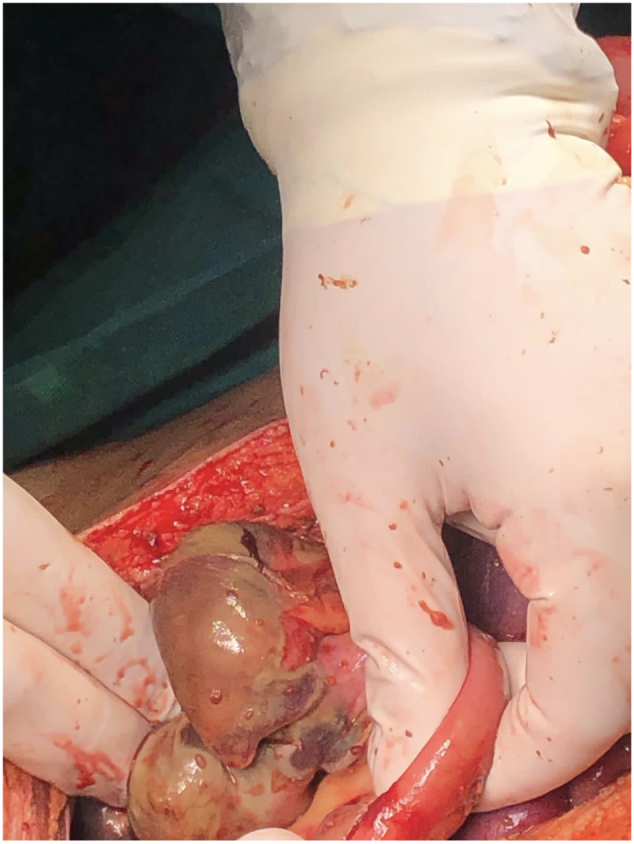
The intraoperative image demonstrates the cecum with evident gangrenous changes, characterized by a darkened, discolored, and necrotic appearance.

A repeat abdominal CT scan (Fig. 3) revealed thickening of the colonic wall, increased density of surrounding mesenteric fat due to inflammation or edema, and mucosal layer enhancement. Hypo-enhancing circumferential wall thickening was noted, primarily involving the ascending colon and extending to the mid-transverse colon. Non-enhancement of the cecal wall, with multiple gas locules at the expected location, suggested bowel ischemia affecting the right colon and possibly extending into the terminal ileum, likely secondary to vasculitis-induced intraluminal thrombosis associated with the patient's known PAN. Large bowel ischemia was suspected, and the patient underwent exploratory laparotomy. Intraoperative findings included gangrenous cecum (Fig. 4), unhealthy bowel from 90 cm proximal to the transverse colon, a perforated ileum, and retroperitoneal hematoma. Despite the patient being in a state of hemodynamic instability with signs of peritonitis, the decision was made to proceed with an extended right hemicolectomy and ileal resection. The patient's vital signs were unstable, and there were signs of systemic infection, including peritonitis, which made it critical to remove the necrotic bowel to prevent further sepsis and septic shock**.** The surgical team prioritized the need for controlling sepsis and improving the patient's hemodynamic stability by resecting the ischemic and gangrenous bowel. The perforated ileum and large ischemic segments in the ascending colon posed a significant risk for further contamination and peritoneal spread of infection. The bowel resection was followed by closure of the transverse colon using a stapler and manual closure of the small bowel, ensuring that the remaining viable bowel was returned to the abdomen.

Histopathological analysis revealed neutrophilic and mononuclear cell infiltration at the base of the intestinal wall, with vasodilatation and congestion. Postoperatively, glucocorticoids were withheld, and the patient received intravenous immunoglobulin (20 g daily for 3 days) and cyclophosphamide (0.2 g IV every other day), alongside antibiotic and nutritional support. The patient's condition gradually improved, and on the 10th postoperative day, prednisone 20 mg daily was added orally.

The patient's abdominal pain resolved, there was no fever, the surgical incision healed well, and there were no gastrointestinal symptoms post-meal. Follow-up laboratory tests, including ESR, CRP, liver and kidney function, and routine blood tests, returned to normal values.

## 3 Discussion

In 1990, the American College of Rheumatology (ACR) established criteria to distinguish Polyarteritis Nodosa (PAN) from other types of vasculitis for research purposes. The committee identified 10 features characteristic of PAN, and a diagnosis requires the presence of at least 3 of these criteria, along with radiographic or pathological confirmation of vasculitis: weight loss of 4 kg or more, livedo reticularis, testicular pain or tenderness, myalgia or leg weakness/tenderness, mononeuropathy or polyneuropathy, diastolic blood pressure >90 mmHg, elevated blood urea nitrogen (BUN) or creatinine levels not linked to dehydration or obstruction, presence of hepatitis B surface antigen or antibody in serum, arteriogram revealing aneurysms or occlusions in the visceral arteries, and presence of polymorphonuclear neutrophils in a biopsy from a small- or medium-sized artery [1].

General Symptoms: Many patients experience anorexia, weight loss, fever, and muscle or joint pain (knees, ankles, elbows, wrists). Renal Manifestations: Vascular nephropathy can lead to variable renal insufficiency, hypertension, and sometimes acute renal failure, which may require dialysis. Angiograms often reveal renal infarcts, stenoses, and microaneurysms. Orchitis: Non-infectious orchitis, indicating testicular artery involvement, is distinctive in PAN and can respond to corticosteroids [6]. Skin Manifestations: Common signs include vascular purpura, nodules, and livedo reticularis, mainly on the legs. Peripheral Vascular Symptoms: These can result in gangrene or Raynaud's phenomenon due to arterial obstruction. Gastrointestinal (GI) Involvement: This is seen in severe PAN cases, especially HBV-related, causing abdominal pain, ischemia, GI bleeding, and sometimes chronic pancreatitis or gallbladder vasculitis [4]. Cardiac Manifestations: Coronary vasculitis can cause left heart failure and supraventricular arrhythmias, though angina is rare. HBV-Related PAN: Typically occurs within a year of hepatitis B infection and shares many PAN symptoms, especially in abdominal and renal involvement. Seroconversion often leads to recovery [5].

Up to 75 % of PAN patients may experience kidney involvement, characterized by arterial stenosis and aneurysms. Typical symptoms include hematuria, proteinuria, new-onset hypertension, and kidney infarction. Angiography may show renal infarctions, multiple narrowings, and microaneurysms. The severity of PAN increases the likelihood of aneurysms, which may rupture, leading to life-threatening complications, such as perirenal hemorrhage and potentially necessitating procedures like embolization or nephrectomy [6]. In our patient, initial vascular CTA suggested a renal aneurysm. After treatment with glucocorticoids and cyclophosphamide, the RAA significantly reduced in size, confirming the diagnosis of PAN complicated by RAA.

Glucocorticoids and cyclophosphamide are the foundation of therapy for PAN. Treatment decisions are primarily guided by the pattern of organ involvement and disease progression. For mild primary PAN, corticosteroids alone are generally effective; prednisone or prednisolone is typically prescribed at an initial dose of 1 mg/kg/day, with a gradual taper as remission is achieved [7,8]. In cases with persistent involvement of critical organs, cyclophosphamide is added to achieve remission [5]. Once remission is induced, a safer immunosuppressive agent, such as azathioprine or methotrexate, is recommended to maintain it [9].

Severe mesenteric artery inflammation can lead to intestinal ischemia, perforation, and bleeding. In extreme cases, ischemic enteritis can cause hematochezia, and full-thickness intestinal wall ischemia might lead to perforation. About 10 % of PAN patients experience GI bleeding or require surgery due to complications [10,11]. The most common issue is small bowel ischemia, while ischemia in the colon or stomach is rare. Small bowel perforation and gastrointestinal bleeding are the most serious symptoms [4].

A poor prognosis was linked to complications such as bleeding, perforation, infarction, and pancreatitis, whereas factors like abdominal pain and cholecystitis did not have a significant impact on outcomes. In a multivariate analysis, proteinuria and gastrointestinal involvement emerged as the primary indicators of poor prognosis [4]. In severe cases of PAN, surgical intervention may become essential for managing complications such as bowel perforation (Table 1) [1,13]. Our patient underwent exploratory laparotomy, revealing a gangrenous cecum, compromised bowel extending from 90 cm distal to the ligament of Treitz to the transverse colon, a perforated ileum, and a retroperitoneal hematoma. Surgical management required resection of the gangrenous bowel, an extended right hemicolectomy, and ileal resection.

**Table 1:** Comparison of two cases of Polyarteritis nodosa with bowel involvement.

Feature	Case one [1]	Case two [13]
Presentation	58 M: Recurrent abdominal pain, lower limb neuropathy, weight loss (>20 kg), testicular pain, new HTN, ↑ESR/CRP, anemia, thrombocytosis.	53 M with RA/HTN on Prednisone 30 mg + MTX 5 mg: 3-day right abdominal pain, obstipation, tachycardic AF (HR 144), surgical abdomen. Labs: WBC 33 × 10^9^/L, CRP 200 mg/L. CT: **sigmoid perforation, free gas/fluid, bowel wall thickening.
Disease type	Polyarteritis Nodosa (PAN) – necrotizing medium-vessel vasculitis; mesenteric ischemia → ileal perforation.	Sigmoid diverticulitis with perforation (CT-confirmed) → PAN by pathology
Response to medical therapy	Partially Steroids + cyclophosphamide improved Symptoms	No pre-hospital improvement on immunosuppression → emergent surgery.
Immunosuppressive treatment	Methylprednisolone (40 → 80 mg IV), cyclophosphamide (0.1 → 0.2 g). - Post-op: IVIG, resumed cyclophosphamide + prednisone taper. - Maintenance: Cyclophosphamide 50 mg (long-term remission).	Pre-op: Prednisone 30 mg/day + MTX 5 mg/day. - Post-op: Steroids tapered, MTX halted (sepsis risk), broad-spectrum antibiotics (meropenem/vancomycin). - Maintenance: Prednisolone 75 mg/day + cyclophosphamide 750 mg/day
Vascular damage	-Renal aneurysms (CTA). - Mesenteric vasculitis → bowel ischemia/perforation.	Mesenteric vasculitis (CT scan)
Surgical intervention	Laparoscopic ileal repair + ileostomy (2018); reversal (2019). Pathology: Vasculitis (neutrophils, vascular congestion).	Hartmann's procedure (sigmoid resection/colostomy). Pathology: medium-size vasculitis
Complications	Peritonitis from perforation	–
Outcome	Sustained remission (5+ years)	colostomy care. No recurrence at 6 months; reversal planned.

## 4 Conclusion

This case illustrates the severe complications of Polyarteritis Nodosa, including RAA rupture and large bowel ischemia, both of which can be life-threatening. Prompt diagnosis and immediate intervention, including angiographic embolization and surgical resection of ischemic bowel, were crucial in managing these complications. This case emphasizes the need for a multidisciplinary approach in severe Polyarteritis Nodosa, integrating close monitoring, rapid imaging, and combined therapy with glucocorticoids and immunosuppressive agents to achieve optimal outcomes and prevent further complications.

## CRediT authorship contribution statement

Ali F. Rabaya, Kareem Ibraheem: Conceptualization, case analysis, manuscript writing, and editing.

Omar Shawer, Mutasem N. Nairat, Sayf Sayes: Data collection, literature review, and manuscript drafting.

Rafiq Salhab, Ali F. Rabaya: Clinical management of the patient, data interpretation, and manuscript revision. All authors have read and approved the final manuscript and agree to be accountable for all aspects of the work.

## Patient consent

Written informed consent was obtained from the patient for the publication of this case report.

## Ethical approval

Ethical clearance was not necessary at our university for a single case report.

## Guarantor

Ali F. Rabaya.

## Funding statement

No sources of funding for this case report.

## Declaration of competing interest

The authors have no conflict of interest to declare.

## Data Availability

The data used to support the findings of this study are included in the article.
